# Supplementary material for: Milestone in predicting core plasma turbulence: successful multi-channel validation of the gyrokinetic code GENE
Source: Nat Commun. 2025 Mar 15;16:2558. doi: 10.1038/s41467-025-56997-2 (PMC11910665; doi:10.1038/s41467-025-56997-2)
Supplement: Supplementary file 1 — Supplementary Information [file 41467_2025_56997_MOESM1_ESM.pdf]

Supplementary Information
Milestone in predicting core plasma turbulence: Successful multi-channel validation of the gyrokinetic code GENE

Klara Höfler[1,2*], Tobias Görler[1], Tim Happel[1], Carsten Lechte[3], Pedro Molina[4,1,5], Michael Bergmann[1], Rachel Bielajew[4], Garrard D. Conway[1], Pierre David[1], Severin S. Denk[4,6], Rainer Fischer[1], Pascale Hennequin[7], Frank Jenko[1], Rachael M. McDermott[1], Anne E. White[4], Ulrich Stroth[1,2], the ASDEX Upgrade Team[1,8]

[1]Max Planck Institute for Plasma Physics, Boltzmannstr. 2, Garching, 85748, Germany.
[2]Technical University of Munich, TUM School of Natural Sciences, Physics Department, James-Franck-Str. 1, Garching, 85748, Germany.
[3]University of Stuttgart, Institute of Interfacial Process Engineering and Plasma Technology, Pfaffenwaldring 31, Stuttgart, 70569, Germany.
[4]Plasma Science and Fusion Center, Massachusetts Institute of Technology, 77 Massachusetts Ave, Cambridge, Massachusetts, 02139, United States of America.
[5]Ecole Polytechnique Fédérale de Lausanne (EPFL), Swiss Plasma Center (SPC), Ecublens, Lausanne, 1015, Switzerland.
[6]General Atomics, General Atomics Court, San Diego, California, 92121, United States of America.
[7]Laboratoire de Physique des Plasmas, Ecole Polytechnique, Rte de Saclay, Palaiseau, 91128, France.
[8]See the author list: H. Zohm et al, 2024 Nucl. Fusion, 10.1088/1741-4326/ad249d.

Supplementary Figure

The figure provided below supports the methods described in the method section.

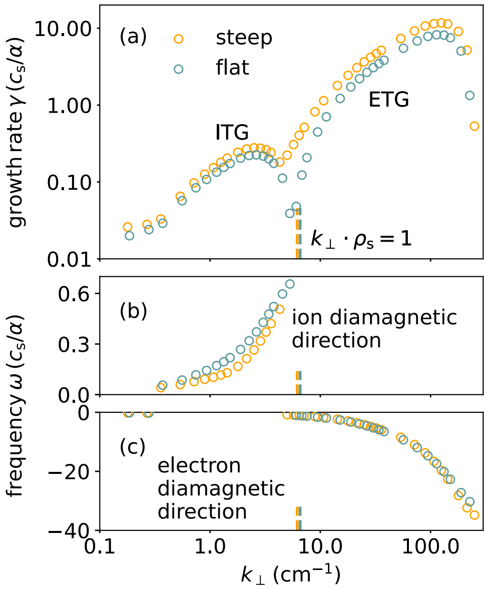

Supplementary Fig. 1 Results from linear GENE runs for both scenarios: growth rates of the fastest growing mode in (a) and frequencies in ion (b) and electron (c) diamagnetic direction, respectively.

Supplementary Table

The table provided below supports the methods described in the method section.

Parameter	steep scenario	flat scenario
Shotnumber	38420	38423
ρ_{pol} (turb. meas.)	0.79	0.79
ρ_{pol} (ECRH deposition)	0.60	0.85
ρ_{tor} (sim.)	0.62	0.62
q	2.01	2.10
shear $\hat{s} = \rho_{\text{tor}}/q \mathrm{d}q/\mathrm{d}\rho_{\text{tor}}$	1.60	1.54
$\alpha = \sqrt{\phi_{\text{sep}}/(\pi B_0)}$ [m]	0.64	0.64
R_0 [m]	1.68	1.68
r/R_0	0.21	0.21
$R_0/\alpha\, \mathrm{d}\rho_{\text{tor}}/\mathrm{d}r$	3.32	3.36
$\alpha/L_{T_{\text{i}}}$	2.36	2.14
$\alpha/L_{T_{\text{e}}}$	3.66	2.84
$\alpha/L_{n_{\text{e}}}$	1.26	0.91
T_{i} [keV]	0.65	0.58
T_{e} [keV]	0.81	0.72
$T_{\text{i}}/T_{\text{e}}$	0.81	0.81
n_{e} [10^{19} m^{-3}]	3.18	3.21
B_0 (B at axis) [T]	-2.53	-2.53
c_{s} [km/s]	196.81	185.54
ρ_{s} [mm]	1.62	1.53
$1/\rho^* = \alpha/\rho_{\text{s}}$	393.70	416.66
β_{ref}	$1.61 \cdot 10^{-3}$	$1.45 \cdot 10^{-3}$
Z_{eff}	1.83	1.75
$\gamma_{E\times B}$ [c_{s}/α]	$4.73 \cdot 10^{-2}$	$4.38 \cdot 10^{-2}$
ν_{c} [c_{s}/α]	$1.08 \cdot 10^{-3}$	$1.37 \cdot 10^{-3}$

Supplementary Table 1 Nominal physics input parameters used in the gyrokinetic flux-tube simulations with the GENE code using ρ_{tor} as the radial coordinate. The logarithmic gradient lengths are thus derived from the experimental profiles as $\alpha/L_X = -\alpha\partial/\partial\rho_{\text{tor}} \ln X$ with $X = T_{\text{i}}, T_{\text{e}}, n_{\text{e}}$. The normalized reference pressure ratio is given in cgs units as $\beta{=}8\pi n_{\text{e}} T_e/B_0^2$, the shearing rate is defined here as $\gamma_{E\times B} = \rho_{\text{tor}}/q\, \partial\Omega_{\text{tor}}/\partial\rho_{\text{tor}}$ with toroidal angular velocity Ω_{tor} and the normalized collision frequency as $\nu_{\text{c}} = \pi \ln\Lambda\, e^4 n_{\text{e}}\alpha/\left(2^{3/2}T_{\text{e}}^2\right)$ with the Coulomb logarithm $\ln\Lambda$.
